# Examining Domestic and Family Violence Typologies Using Queensland Police Data: Differences in Risk of Harm

**DOI:** 10.1177/08862605261447033

**Published:** 2026-05-26

**Authors:** Rebecca A. Howard, Mark R. Kebbell

**Affiliations:** 1 Griffith University, Brisbane, QLD, Australia

**Keywords:** domestic family violence, intimate partner violence, violence against women, intimate partner homicide, offender typologies, police, cluster analysis

## Abstract

Identifying domestic and family violence (DFV) offender typologies can aid risk assessment and intervention. However, the current body of literature is limited in its inconsistent, resource-intensive methodologies and resultingly small sample sizes. The current study explores Holtzworth-Munroe and colleagues’ intimate partner violence typologies in a large-scale sample of 10,956 male Queensland DFV offenders. We focused on a generality of offending measures, using police records of offending histories. Hierarchical cluster analysis with Ward’s linkage clustered perpetrators based on offending in 16 categories. The four-cluster solution comprised homicidal, generally violent/antisocial (GVA), low-level antisocial (LLA), and family-only offenders. Aligning with previous literature, family-only and GVA offenders presented at opposing ends of a continuum of antisociality. Family-only demonstrated the lowest-level offending, largely restricted to violence within the family. GVA displayed a variety of severe and frequent offending, including intra and extrafamilial violence. LLA clustered between the two, showing a similar yet less severe pattern to GVA. The novel homicidal subtype suggested that offenders who escalate to homicide may not fit within general DFV typologies. A Kruskal-Wallis nonparametric test with Dunn’s pairwise comparisons examined differences in harm caused, identifying cluster risk-levels. All clusters differed significantly, with homicidal offenders displaying highest harm levels, followed by GVA, LLA, and family-only, respectively. This showed typologies of differing risks could be identified by police to some degree, with implications for future research and practice. Research focusing on generality of violence could identify typologies at a larger scale more consistently, and provide information applicable to policing. Grouping at a policing level could support identifying high-risk offenders and implementing tailored interventions.

## Introduction

Domestic and family violence (DFV) is a pressing global issue, occurring in various socioeconomic, religious, and cultural groups (World Health Organization [WHO], 2012). DFV – and in particular, intimate partner violence (IPV) – is the most widespread form of violence against women, and is perpetrated primarily by men (Caldwell et al., 2012; WHO, 2021). Deaths are not uncommon, with IPV homicide occurring approximately every 10 days in Australia (Australian Institute of Health and Welfare, 2024), and global domestic femicides averaging 140 deaths per day (United Nations, 2024). Given the diversity of offender populations, identifying meaningful groupings can support harm-reduction strategies (Petersson & Strand, 2020; Petersson et al., 2019).

Categorising DFV offenders based on common characteristics aids understanding factors that underpin offending, prediction of risk-level, and developing tailored interventions. DFV involves a broad range of offenders with various relationships to their victims, including parent-on-child, child-on-parent, sibling, IPV, and more. IPV is particularly widespread and is responsible for most reported DFV (Queensland Courts, 2025). Because of this, much of the foundational typological literature and studies testing these theories focus on IPV.

Holtzworth-Munroe and Stuart (1994) examined male IPV literature for underlying patterns and defined three distinct ‘types’ of batterers: family-only, dysphoric/borderline, and generally violent/antisocial (GVA). These typologies were classified using three dimensions: severity of violence, generality of violence, and psychopathology. Family-only offenders theoretically scored lowest on all dimensions. GVA scored the highest. Dysphoric/borderline displayed inflated psychopathology and moderate to high severity violence. Holtzworth-Munroe et al. (2000) found support for the hypothesised three subtypes using a community sample of men, with the addition of a fourth subtype, low-level antisocial (LLA). LLA clustered between family-only and GVA, showing similar, yet less severe, offending patterns to GVA offenders.

Identification of offender subtypes can aid in understanding risk factors for violence and areas to target in interventions. For example, family-only offenders often experienced more guilt, leading to compliance and behavioural improvements with any level of intervention (Cantos et al., 2019; Petersson & Strand, 2020), while GVA offenders displayed antisocial attitudes, requiring more motivationally-driven interventions (Cantos et al., 2015; Petersson & Strand, 2020). These typologies are widely cited and influential in IPV literature (Alexander & Johnson, 2023; Wangmann, 2011), and other typological research has notably similar findings (Cavanaugh & Gelles, 2005; Holtzworth-Munroe & Meehan, 2004; Wangmann, 2011).

Cavanaugh and Gelles (2005) synthesised key typological theories and suggested that most research categorises offenders along a continuum of low to high risk. Low-risk resemble family-only offenders, while high-risk include GVA and dysphoric/borderline offenders. They suggested further subtyping using severity and frequency of violence, psychopathology, and criminal history, to discriminate more detailed subtypes like dysphoric/borderline offenders who are characterised largely through personality and psychopathology. Critically, Cavanaugh and Gelles (2005) argued the utility of typologies, with different offenders requiring tailored interventions.

### Validating and Measuring Offender Typologies

Although attempts to validate Holtzworth-Munroe and Stuart (1994) and Holtzworth-Munroe et al. (2000) have seen some inconsistent results, likely due to sampling and methodological differences, the typology is generally supported (Alexander & Johnson, 2023). Wray et al.’s (2015) two-step cluster analysis using self-report and court conviction data found all three Holtzworth-Munroe and Stuart (1994) subtypes. Critically, findings aligned with the hypothesised subtype frequency of offending, with family-only committing the least offences and GVA committing the most. However, as their sample included court-referred offenders, it may be biased towards more severe offenders, with the least violent, family-only perpetrators, being underrepresented.

Petersson and Strand’s (2020) systematic review of family-only perpetrators included samples from clinical, community, correctional, and police settings. They found the literature largely aligned with Holtzworth-Munroe and Stuart’s (1994) predictions; family-only offenders committed less violent crimes and displayed fewer psychopathologies. GVA appeared in contrast, showing higher severity IPV and general antisocial behaviours.

Studies employing cluster analyses revealed offenders matching Holtzworth-Munroe and Stuart’s (1994) original types (Stoops et al., 2010; Walsh et al., 2010), as well as Holtzworth-Munroe et al.’s (2000) LLA offenders, clustering between family-only and GVA (Graña et al., 2014; Weber & Bouman, 2020), and showing moderate recidivism risk (Llor-Esteban et al., 2016). Family-only and GVA subtypes are particularly well supported. Studies classifying offenders with cluster analyses (Graña et al., 2014; Stoops et al., 2010; Walsh et al., 2010; Weber & Bouman, 2020; Wray et al., 2015) as well as separating offenders based on generality of violence (Boyle et al., 2008; Petersson & Strand, 2017; Petersson et al., 2019; Theobald et al., 2016) found these distinct types.

Findings are less consistent for dysphoric/borderline offenders. Cluster analyses using subjective measures of personality and psychopathology have identified them (e.g., Llor-Esteban et al., 2016; Stoops et al., 2010; Walsh et al., 2010; Weber & Bouman, 2020; Wray et al., 2015). However, as dysphoric/borderline offenders’ defining characteristics involve personality traits and psychopathology, studies using objective measures like court and conviction data cannot discern this subtype (e.g., Boyle et al., 2008; Petersson & Strand, 2017; Petersson et al., 2019; Theobald et al., 2016). Measures of personality, psychopathology, and attitudes are most likely to reveal this subtype. However, social desirability biases and positive attitudes towards crime can lead offenders to downplay their actions, making psychopathology less obvious (Wray et al., 2015). This raises methodological questions and criticisms, and more consistent methodologies are needed to reliably determine classifications (Alexander & Johnson, 2023).

With this in mind, utilising *generality of violence* – the range of offences perpetrators commit – as the key determinant in classification may provide more consistency. Indeed, researchers classifying IPV offenders this way consistently found subtypes resembling family-only and GVA (Boxall et al., 2015a; Boyle et al., 2008; Petersson et al., 2019; Theobald et al., 2016), as well as LLA clustering between them (Graña et al., 2014; Llor-Esteban et al., 2016; Weber & Bouman, 2020). Holtzworth-Munroe et al. (2000) theorised family-only, LLA, and GVA subtypes could be conceptualised along a continuum of antisociality, from low (family-only) to high (GVA). However, identification of dysphoric/borderline offenders relies on subjective measures of psychopathology (such as clinical interviews), which can be biased and difficult to gather on a large scale. Considering increased consistency found with measures of violence generality, offenders placed along the antisociality continuum may be readily identified with conviction history alone. This is more feasible for large-scale research, and could aid responses at policing levels.

### Response and Prevention Implications: Utilising Offender Typologies to Predict Harm

Appropriate classification could assist in the prediction of risk and recidivism. Cavanaugh and Gelles (2005) found typological approaches consistently identified different groups of offenders, with higher-risk groups responsible for more harmful offences. Peters et al.’s (2023) latent class analysis of 244 IPV offenders found significant differences in recidivism for family-only and GVA men. Family-only had a lower measured reoffending risk, and around half the recidivism rate of GVA. GVA also displayed more severe violence and higher harm in their offending. Researchers classifying perpetrators based on scores on the structured Brief Spousal Assault Form for the Evaluation of Risk (B-SAFER) tool found groupings resembling Holtzworth-Munroe et al.’s (2000) subtypes. Harm risk matched theorised levels, with family-only scoring lowest, GVA highest, and LLA showing moderate risk (Serie et al., 2015; Thijssen & de Ruiter, 2011).

Studies found GVA offenders committed more frequent and severe abuses. This includes homicide related offences, serious assaults, and sexual and psychological IPV (Ouellet et al., 2016). They scored higher on personality and attitude measures that maintained violence and increased reoffending risk (Theobald et al., 2016), and displayed higher recidivism over longer periods of time (Petersson & Strand, 2017). Additionally, Dixon et al. (2008) classified 90 men incarcerated for partner homicide in England based on Holtzworth-Munroe and Stuart’s (1994) typology, with 85% of offenders matching GVA or dysphoric/borderline subtypes. In contrast, community-based and clinical samples typically found family-only to be most common (e.g., Graña et al., 2014; Holtzworth-Munroe & Stuart, 1994; Petersson & Strand, 2020; Walsh et al., 2010). Dixon et al.’s (2008) overrepresentation of GVA and dysphoric/borderline offenders demonstrates these subtypes cause higher harm and are higher risk.

By identifying offender subtypes, services could tailor interventions to better address perpetrators based on harm and likelihood of reoffending (Boxall et al., 2015b; Cavanaugh and Gelles, 2005; Peters et al, 2023), and even adjust the weighting of risk tool items according to offender typology to improve predictive validity (Aguilar Ruiz & González-Calderón, 2022). For example, family-only offenders consistently displayed lower risk scores, higher compliance with interventions, and made more progress during treatment compared to GVA (Aguilar Ruiz & González-Calderón, 2022; Petersson & Strand, 2020; Stoops et al., 2010). Their characteristics like remorse and more positive attitudes towards women can be utilised in interventions (Cantos et al., 2019; Petersson & Strand, 2020). In contrast, GVA consistently showed the highest risk for harm and recidivism, as well as the highest intervention drop-out and noncompliance, and may require more significant justice-system responses addressing substance use, unemployment, aggression, and general antisocial characteristics (Aguilar Ruiz & González-Calderón, 2022; Cantos et al., 2015, 2019; Holtzworth-Munroe & Meehan, 2004; Llor-Esteban et al., 2016; Petersson & Strand, 2020; Petersson et al., 2019; Stoops et al., 2010). Importantly, GVA perpetrators who completed treatment sometimes showed more improvements compared to other subtypes (Cantos et al., 2019; Huss & Ralston, 2008). This may be due to higher baseline violence, but reveals that GVA perpetrators can be responsive to treatment. These differing risk and intervention findings demonstrate the utility of classifying offenders at policing levels.

### Current Study

Existing typologies rely on small samples due to complex, time-intensive measures; large-scale administrative data remain under explored. The present study examined typologies using a sample of DFV offenders from Queensland Police Service (QPS) data. This aimed to extend upon previous literature by classifying offenders on a larger scale, using comprehensive offending information from the Queensland Police Records and Information Management Exchange (QPRIME) database. The data did not allow for identification of offender-victim relationships, so IPV could not be differentiated from other DFV offences. However, as 70% of DFV cases reported to police in Queensland involve intimate partners (Queensland Courts, 2025), and most influential typological literature focuses on IPV, we chose to examine the data with IPV literature in mind.

Cluster analysis grouped individuals into clusters before further analyses determined between-group harm differences. Police data were used to determine if practical categorisation could be conducted at a policing level, to assist early response and risk identification. We utilised measures of offending based on police identified criminal occurrences to address previous literatures’ methodological issues, while also expanding the literature by examining whether valid subtypes can be identified without in-depth analysis of personality and psychopathology. This would allow for increased sample sizes and consistency going forward, as these methods reduce the need for problematic subjective measures and one-on-one contact with perpetrators. Because of this, it is expected that subtypes characterised largely through personality and psychopathology (i.e., dysphoric/borderline) will not be identifiable. Based on the literature, it was hypothesised that:

H1: A cluster of offenders will resemble family-only, showing limited criminal occurrences, largely restricted to within family DFV offences;
H2: Another cluster will resemble GVA, with more criminal occurrences including DFV, more general extrafamilial violence, and additional non-violent crimes like substance use offences;
H3: The GVA cluster will be associated with more harm than the family-only cluster.

## Method

### Sample

A sample of DFV offenders (*N* = 13,103) was drawn from QPS data, and female (*n* = 2,143), unknown gender (*n* = 2), and under 14-year-old (*n* = 2) offenders were excluded to reduce the sample to our population of interest based on the previous literature. The final sample comprised 10,956 male DFV offenders. Although there is no generally accepted guideline for required sample size to obtain sufficient power, Sarstedt and Mooi (2019) suggest a minimum of 10 times the number of participants as variables in the cluster analysis is required in ideal cases with equally sized clusters. They recommend a sample size of 70 times the number of variables analysed to substantially improve the cluster solution if distinctions between clusters are harder to find or clusters are unequal. Although the data used are sufficient, it is noted that a minimum of 1,120 participants should be analysed to replicate this study, as clusters are likely to be unequal in size. Regarding Indigeneity, 1,469 (13.4%) of the sample were identified as Aboriginal and/or Torres Strait Islander, 3,019 (27.6%) were not Indigenous, and 6,468 (59%) cases did not include this information. Mean age at index offence was 33.96 (*SD* = 10.14), ranging from 14 to 89.

### Measures

#### Demographic Measures

Gender and age at index offence were utilised to limit the sample to the population of interest. Age and Indigeneity examined the general sample demographic characteristics.

#### Offence Related Variables

Offence category variables were created based on Australian and New Zealand Standard Offence Classifications (Australian Bureau of Statistics, 2023), as explained in *Data Source and Procedures.* The variables contained count data, detailing the number of offences within classifications committed by individual perpetrators. Counts of offences were based on police data on ‘solved’ crimes. This refers to offences that, based on police judgement, are reasonably believed to have been committed by the offender. Because of this, offences included were more extensive than court or conviction data, as occurrences that did not result in convictions were listed. Table 1 displays the 16 offence category variables analysed in the cluster analysis, and the individual offences they included. To conduct the cluster analysis, offence category variables were standardised using *z*-scores, so differing ranges of offence counts across types did not unduly impact the solution. This was important as the prevalence of offending varied greatly by offence type. Counts of the least prevalent type, homicide, ranged from 0 to 4 per offender, while the most prevalent, burglary/theft, ranged from 0 to 156. The *z*-Score transformation converted this count data into a standard scale wherein scores indicated how far an individual was from the mean. Finally, variables quantifying perpetrators’ total numbers of offences and total numbers of victims explored median offending frequency and generality for the overall sample and each cluster.

**Table 1.** Offence Categories and Included Offences.

Offence Type	Offences
Homicide	Murder Attempted murder Manslaughter Manslaughter/driving causing death
Assault	Serious assault resulting in injury Serious assault not resulting in injury Common assault
Sexual	Aggravated sexual assault Non-aggravated sexual assault Non-assaultive sexual offences Non-assaultive sexual offences against a child Child pornography offences
Harm or endangerment	Abduction and kidnapping Deprivation of liberty/false imprisonment Stalking Neglect/ill-treatment of person under care Other dangerous/negligent acts endangering persons
Dangerous operation of motor vehicle	Dangerous/negligent operation/driving of a vehicle Motor vehicle incident low-level Motor vehicle incident low-level with injury Motor vehicle incident high-level Motor vehicle incident high-level with injury
Robbery, blackmail, extortion	Non-aggravated robbery Aggravated robbery Blackmail/Extortion
Burglary/theft	Unlawful entry with intent/burglary/break and enter Theft of motor vehicle Theft of motor vehicle parts/contents Theft from a person excluding by force Theft from retail premises Theft except motor vehicles nec. Receive/handle proceeds of crime Theft and related offences nfd.
Fraud, deception, and related	Obtained benefit by deception Counterfeiting of currency Dishonest conversion nfd. Misrepresentation of professional status Forgery of documents Possess equipment to make false/illegal instrument Offences against privacy Fraud, deception, and related offences nfd.
Drug	Possess and/or use illicit drugs Manufacture/cultivate illicit drugs Deal/traffic illicit drugs non-commercial quantity Deal/traffic illicit drugs commercial quantity Import/export illicit drugs
Weapons	Sell, possess, and/or use prohibited weapons/explosives Unlawfully obtained/possess regulated weapons/explosives
Property	Property damage by fire/explosion Property damage nec. Property damage nfd. Graffiti
Public order, health, and safety	Criminal intent Trespass Offensive behaviour Disorderly conduct Public order offence Regulated public order offence Offences against public order sexual standards
Substance use and gambling	Liquor and tobacco offences Betting and gambling offences
Traffic/vehicle regulatory	Exceed prescribed content of alcohol/other substance limit Drive while licence disqualified/suspended Registration offence Regulatory driving offence
Justice procedures and government	Subvert course of justice Resist or hinder police officer or justice official Escape custody Breach of bail Breach of community service order Breach of community-based order Court breach Bribery involving government officials Offences against justice procedures, government security, government nec.
Domestic violence and related	Domestic violence application Breach of domestic violence order Index offence

*Note.* nfd. = not further defined; nec. = not elsewhere classified.

#### Offender Harm Index

An offender harm index quantifying perpetrators’ harm caused in relation to all offences was analysed to determine differences between clusters in risk level. The harm index utilised the Western Australian Crime Harm Index (WACHI), which allocates offences numerical values based on median days of imprisonment first-time offenders received for that offence (House & Neyroud, 2018). For example, least harmful offences like unlicensed vehicle offences were allocated a value of 1, while murder, the most harmful, earned a value of 6,023. An individual’s total harm score was calculated by summing harm values for each offence committed.

### Data Source and Procedures

A retrospective cohort study with a longitudinal design was conducted using secondary QPS data drawn from the QPRIME database and stored in a secure university research facility. Due to its sensitive nature, ethical and confidentiality requirements restrict this data from being shared. Ethical approval from the university’s Human Research Ethics Committee was obtained in 2022 to use the data, and a variation was approved to include the present study and researchers on April 23, 2024 (GU Ref No: 2022/181). The data contained a total population sample of all offenders in contact with QPS for a DFV offence from January 1, 2016 until December 31, 2017; and included information of this offence as well as lifetime offending data committed before or after the index offence, reported to police between 2008 and 2022. The de-identified dataset described information about each offender, including: gender, indigeneity, age, type and quantity of offences committed, and number of victims perpetrated against.

The initial dataset containing 13,103 offenders had previously been cleaned to remove cases with significant missing or erroneous data. As previous literature focused on males, with contention regarding whether the same subtypes were appropriate for women (Wangmann, 2011), all female offenders (*n* = 2,143), as well as two offenders missing gender information were removed, reducing the sample to 10,958. All offenders aged under 14 at time of index offence were removed (*n* = 2), bringing the final sample to 10,956 cases. This was done as young juvenile offenders typically have differing offender-victim relationships (with IPV less common) and contextual factors relating to their offending, making them map differently onto typologies (Freeman, 2018). However, as Holtzworth-Munroe et al.’s (2000) typologies also showed different age-related patterns (e.g., GVA offending in adolescence more commonly than family-only), we did not want to completely exclude adolescent offenders. The age cutoff of 14 was chosen as previous research commonly examines juveniles from around this age and onwards, and finds IPV begins to increase at around this age (e.g., Foshee et al., 2009; 2013; Heinze et al., 2018).

The dataset contained count data for offences in addition to the index offence committed by each perpetrator along 76 individual offence variables. These were combined into 16 offence category variables based on the Australian and New Zealand Standard Offence Classifications (Australian Bureau of Statistics, 2023; see Table 1). This was done to simplify the cluster analysis procedure, reducing the processing power needed to run the analysis. As data did not include detailed descriptions of offences, determining whether offences were related to DFV was sometimes difficult. To create the *Domestic Violence and Related* offence category, offences directly related to DFV (i.e., domestic violence application and breach of violence order offences) were merged with an additional one offence added to account for the index offence. Although this does not capture all DFV offences, which may fall under groupings such as assault, sexual, homicide, and harm or endangerment offences, it separates offences known to be related to DFV and allows for inclusion of the index offence as a DFV offence.

## Results

### Analytical Plan

Analyses were conducted using IBM’s Statistical Package for Social Sciences (SPSS) Version 28. Graphs to aid visualisation of findings were generated in Microsoft Office 365 Excel. A hierarchical cluster analysis with Ward’s linkage clustered perpetrators based on quantity and range of offending using the 16 offence category variables, standardised using *z*-scores, so different ranges of scores did not impact the solution. Hierarchical clustering was used over methods such as *k*-means clustering as it allowed for determining the most appropriate number of clusters, and Ward’s linkage was chosen over other linkage alternatives as it is best for making clusters of reasonably equal sizes and variances by linking cases in an order that minimises the error sums of squares (Ward, 1963), and was found to be most reliable and one of the most common methods for classifying DFV perpetrators (Holtzworth-Munroe et al., 2000; Huss & Ralston, 2008; Llor-Esteban et al., 2016; Weber & Bouman, 2020).

Following recommendations made by Yim and Ramdeen (2015), an examination of the dendrogram and agglomeration schedule coefficients determined the best cluster solution. The dendrogram (Appendix A) visually represented the hierarchical relationship between offenders. A cluster solution was found by choosing an appropriate point to ‘slice’ down the diagram vertically, separating clusters in an area that maximised differences while keeping the number of groups theoretically meaningful and simple to interpret. Coefficients from the final 20 stages of the agglomeration schedule (representing solutions ranging 1–20 clusters) were plotted on a line graph (Appendix B). The horizontal axis displayed the stage in agglomerative clustering, while the vertical axis displayed agglomerative coefficients representing the dissimilarity between clusters at the corresponding stage. The optimal number of clusters was examined by finding the ‘elbow’ point wherein coefficients begin to follow a smoother decline, indicating more similarity among clusters.

After finding an appropriate cluster solution, a Kruskal-Wallis nonparametric test with eta-squared effect size analysed differences in harm index scores between clusters. This was used to validate the cluster solution by examining whether significantly different groupings were created. This test was used in place of an analysis of variance (ANOVA) as assumptions of normality and homogeneity of variance were not met, and data distributions were not improved following a square root transformation. Post hoc analyses examining differences were conducted using Dunn’s pairwise comparisons adjusted with Bonferroni’s correction and *r* effect sizes.

Descriptive statistics were generated to examine average offending patterns across the whole sample, as well as for each cluster. The variables examined included: all offence categories, total number of offences, total number of victims, and offender harm. Medians and interquartile ranges (IQR) were used as all variables were positively skewed, with outlying cases at the higher ends.

### Descriptives

Table 2 presents medians and interquartile ranges for different offence categories among the entire sample to provide context on the overall average offending pattern. Additionally, minimum and maximum scores for each variable are displayed to show offending on the extreme ends, as many outliers were found with higher end offending. The total number of offences committed by each perpetrator ranged from 1 to 362 (Mdn = 21, IQR = 9, 41). The median number of unique victims per perpetrator was 2 (IQR = 0, 4, range: 0–118). There was large variability in harm, with scores ranging from 4 to 18,216.58 (Mdn = 132.73, IQR = 47.45, 363.26).

**Table 2.** Offending Descriptives of Entire Sample.

Variables of Interest	Mdn	IQR	Min	Max
Offence category variables				
Homicide	0	0, 0	0	4
Assault	1	0, 2	0	45
Sexual	0	0, 0	0	16
Harm or endangerment	0	0, 0	0	14
Dangerous operation of motor vehicle	0	0, 0	0	12
Robbery, blackmail, extortion	0	0, 0	0	12
Burglary/theft	1	0, 4	0	156
Fraud, deception, and related	0	0, 0	0	96
Drug	2	0, 5	0	66
Weapons	0	0, 0	0	10
Property	0	0, 1	0	118
Public order, health, and safety	1	0, 3	0	74
Substance use and gambling	0	0, 0	0	81
Traffic/vehicle regulatory	3	1, 7	0	58
Justice procedures and government	2	0, 7	0	78
Domestic violence and related	5	3, 8	1	43

*Note.* IQR = interquartile range.

### Cluster Analysis

The hierarchical cluster analysis indicated a four-cluster solution best represented the sample. Table 3 displays clusters’ medians and interquartile ranges for all offence categories, total number of offences, and total number of victims. Clusters contained offenders in a wide range of age groups at the index offence, with all groups including young adult, middle-aged, and older offenders, and all apart from cluster 1 including juvenile offenders.

**Table 3.** Four Cluster Solution: Medians and Interquartile Ranges for All Variables of Interest.

Variables of Interest	Cluster 1 (*n* = 71)	Cluster 2 (*n* = 1,235)	Cluster 3 (*n* = 4,462)	Cluster 4 (*n* = 5,188)
	Mdn (IQR)	Mdn (IQR)	Mdn (IQR)	Mdn (IQR)
Offence category variables				
Homicide	1 (1, 1)	0 (0, 0)	0 (0, 0)	0 (0, 0)
Assault	2 (0, 4)	1 (0, 3)	1 (0, 3)	0 (0, 1)
Sexual	0 (0, 0)	0 (0, 0)	0 (0, 0)	0 (0, 0)
Harm or endangerment	0 (0, 1)	0 (0, 1)	0 (0, 1)	0 (0, 0)
Dangerous operation of motor vehicle	0 (0, 0)	0 (0, 1)	0 (0, 0)	0 (0, 0)
Robbery, blackmail, extortion	0 (0, 0)	`	0 (0, 0)	0 (0, 0)
Burglary/theft	1 (0, 5)	10 (3, 26)	2 (0, 5)	0 (0, 1)
Fraud, deception, and related	0 (0, 0)	0 (0, 3)	0 (0, 0)	0 (0, 0)
Drug	2 (0, 6)	9 (4, 15)	3 (0, 6)	0 (0, 2)
Weapons	0 (0, 0)	1 (0, 2)	0 (0, 0)	0 (0, 0)
Property	1 (0, 3)	1 (0, 4)	1 (0, 2)	0 (0, 0)
Public order, health, and safety	1 (0, 4)	2 (0, 4)	2 (0, 4)	0 (0, 1)
Substance use and gambling	0 (0, 0)	0 (0, 0)	0 (0, 1)	0 (0, 0)
Traffic/vehicle regulatory	3 (1, 6)	8 (4, 12)	5 (2, 8)	2 (0, 4)
Justice procedures and government	5 (1, 10)	10 (5, 16)	5 (2, 10)	1 (0, 2)
Domestic violence and related	5 (3, 8)	6 (4, 9)	7 (4, 11)	3 (2, 5)
Total number of offences	35 (14, 48)	64 (44, 90)	35 (23, 50)	10 (5, 17)
Total number of unique victims	4 (2, 8)	7 (3, 15)	3 (1, 6)	1 (0, 1)

Cluster 1 (*n* = 71) was characterised primarily by homicidal offences, with all offenders committing at least one offence in this category (range: 1–4). They also displayed highest medians for assaults, and moderate medians for burglary/theft, drug, property, public order, health, and safety, traffic/vehicle regulatory, justice procedures and government, and domestic violence and related offences. They showed elevated medians for total number of offences and victims compared to that of the whole offender population. They had the second oldest average age at index offence (*M* = 34.87, *SD* = 11.08, range: 19–69). Displaying the highest harm score of all clusters (Mdn = 3,277.20, IQR = 2,572.24, 6,610.47), this group was responsible for far more harm than any other cluster. Comprising only 0.65% of the overall sample, this was the smallest cluster in the solution.

Cluster 2 (*n* = 1,235) was characterised by the highest number of general offending, with moderate to high medians for the largest variety of offences. This consisted of the highest median scores for burglary/theft, drug, weapons, traffic/vehicle regulatory, and justice procedures and government offences. Additionally, they displayed elevated medians for property, public order, health, and safety, and domestic violence and related offences compared to the whole offender population. Cluster 2 had the youngest average age at index offence (*M* = 29.30, *SD* = 8.29, range: 15–62). This cluster showed the highest median scores for total number of offences and total number of victims, and the second highest harm score (Mdn = 461.12, IQR = 221.54, 1,219.60).

Cluster 3 (*n* = 4,462) showed a similar but less severe pattern of offending to cluster 2, with elevated median scores for burglary/theft, drug, property, public order, health, and safety, traffic/vehicle regulatory, justice procedures and government, and domestic violence and related offences compared to the overall population. Medians were consistently lower than cluster 2, apart from public order, health, and safety offences which were equal, and domestic violence and related offences, in which cluster 3 had the highest. They had the second youngest age at index offence (*M* = 32.32, *SD* = 9.07, range: 14–87). Compared to the total population, they displayed elevated medians for total number of offences, total number of victims, and harm. Their median harm score (Mdn = 273.10, IQR = 141.81, 641.23) was lower than clusters 1 and 2, but higher than cluster 4.

Cluster 4 (*n* = 5,188) was characterised by consistently low levels of offending, with offending largely restricted to domestic violence and related, traffic/vehicle regulatory, and justice procedures and government offences. This group displayed the lowest median scores for all offence categories, total number of offences, total number of victims, and harm than any other cluster and the whole offender population. They were the oldest on average at index offence (*M* = 36.47, *SD* = 10.71, range: 15–89). Their median harm index score was 48.38 (IQR = 19.74, 96.15).

### Harm Index Differences

The Kruskal-Wallis nonparametric test indicated a significant difference in harm index mean rank scores of 10,843.54 (cluster 1), 8,428.95 (cluster 2), 7,299.60 (cluster 3), and 3,136.47 (cluster 4); *H*[3, *N* = 10,956] = 5,602.90, *p* < .001, η^2^ = .51. This was a large effect (B. H. Cohen, 2008), with cluster membership accounting for 51% of variability in harm. Post hoc analyses using Dunn’s pairwise comparisons with Bonferroni corrections found significantly different harm scores between all cluster combinations. Cluster 1 displayed a significantly higher mean rank to cluster 2 (*z* = 6.26, *p* < .001, *r* = .17), cluster 3 (*z* = 9.37, *p* < .001, *r* = .14), and cluster 4 (*z* = 20.39, *p* < .001, *r* = .28), indicating cluster 1 was responsible for significantly more harm than all other clusters. These were all small to moderate effects (J. Cohen, 1992). There was a significant, small to moderate difference in harm mean ranks between clusters 2 and 3 (*z* = 11.11, *p* < .001, *r* = .15), with cluster 2 displaying higher harm. Additionally, cluster 2 showed significantly higher harm to cluster 4 (*z* = 52.85, *p* < .001, *r* = .66), with a large effect size. Finally, cluster 3 showed a significantly higher harm index mean rank compared to cluster 4 (*z* = 64.47, *p* < .001, *r* = .66), with a large effect size.

## Discussion

The present study extended typological literature by examining DFV perpetrator classifications at a large scale with police data. The research aimed to examine whether Holtzworth-Munroe et al.’s (2000) IPV typologies were applicable to a Queensland DFV offender sample, and readily identifiable at the policing level. It also aimed to examine whether resulting typologies differed significantly in harm and could therefore be utilised in risk assessment and response efforts. The hierarchical cluster analysis was hypothesised to reveal at least two distinct clusters, with offending patterns resembling Holtzworth-Munroe and Stuart’s (1994) family-only and GVA offenders. (H1) The family-only cluster was hypothesised to display lower offending, with occurrences largely restricted to within-family DFV. (H2) The GVA cluster was hypothesised to display more extensive and frequent offending, including intra and extrafamilial violence, and general offending such as illicit drug offences. Additionally, it was hypothesised that (H3) GVA would be responsible for more harm compared to family-only.

Results of the cluster analysis supported Hypotheses 1 and 2. A four-cluster solution best fits the data, with two clusters resembling family-only and GVA offenders. Cluster 1 comprised less than 1% of the overall sample, and was characterised as homicidal, containing offenders who all committed at least one homicide related offence. This subtype displayed the highest harm index scores, demonstrating a high impact on the overall sample’s harm despite their rarity. Cluster 2 comprised 11% of the sample and matched Holtzworth-Munroe and Stuart’s (1994) GVA offenders. This subtype displayed the highest numbers of offences and victims, consistently high median scores in the largest variety of offences, and were the youngest group on average. Cluster 3 contained 41% of the sample, and generally showed similar yet less severe offending patterns to GVA, so was labelled LLA in line with literature (Holtzworth-Munroe et al., 2000). Finally, cluster 4 comprised 47% of the sample, and matched the older age and more limited offending patterns of previous literature’s family-only offenders (Holtzworth-Munroe & Stuart, 1994; Petersson & Strand, 2020). This subtype demonstrated consistently low-level offending, limited diversity in offences with most pertaining to DFV, and offending against few victims.

The Kruskal-Wallis nonparametric test with Dunn’s pairwise comparisons determined all clusters differed significantly from each other in harm, with homicidal responsible for most harm, followed by GVA, LLA, and family-only, respectively. This supported Hypothesis 3. Overall, findings supported all hypotheses and aligned with previous literature, identifying family-only, LLA, and GVA offenders. The additional homicidal offender cluster demonstrated a novel finding, with implications for future research and offender response services.

### Cluster 1: Homicidal Offenders

Cluster 1 was characterised as homicidal, as all offenders in this subtype committed at least one homicide related offence, and only two outside of this cluster committed homicide offences. Offenders in this category displayed high levels of assaults and moderate levels, similar to overall sample medians, of a range of general and DFV crimes. Regarding these offences, homicidal offenders demonstrated lower median offending compared to GVA and LLA, but higher medians compared to family-only. They were responsible for significantly more harm than all other clusters, suggesting they are higher-risk. This cluster was an unexpected and novel finding.

Homicidal offenders could represent high-end outlying GVA offenders, reflecting the top percentage of GVA offenders who escalate to homicide. This is evidenced by the broad range of other offences present in this subtype. However, this group would be expected to commit higher levels of all offences like the GVA subtype, as GVA is characterised primarily by generalist offending (Boxall et al., 2015a; Holtzworth-Munroe & Stuart, 1994; Petersson et al., 2019). Lower general offending compared to GVA and LLA may be explained by incarceration subsequent to the homicidal offence, suppressing future offending, however the data did not allow investigation of this.

Another possible explanation is homicide offenders may be less predictable compared to other offenders. They may not match other classifications, or may come from multiple subtypes, with all offenders having potential to escalate to homicide. Research classifying intimate partner homicide offenders have found diverse groups, with subtypes differing in personality, psychopathology, and offending patterns (Kivisto, 2015; Péloquin et al., 2024; Vignola-Lévesque & Léveillée, 2021). Kivisto (2015) reviewed intimate partner homicide literature and proposed a typology of offenders ranging from mentally ill offenders with no history of IPV, to chronic batterers with characteristics similar to GVA offenders. Additionally, Péloquin et al.’s (2024) latent profile analysis on intimate partner homicide offenders determined five distinct profiles, ranging from offenders with no previous criminality, offenders with low or moderate IPV histories like family-only and LLA subtypes, to those with extensive histories of general violence similar to GVA offenders. In this case, homicide offenders may be harder to identify and predict among general IPV offender populations, and require investigation into unique risk factors and typologies.

A combination of these speculations likely explains the sample’s presence of homicidal offenders. Dixon et al.’s (2008) identified an overrepresentation of GVA offenders in a homicidal IPV offender sample compared to other samples, suggesting this subtype is more likely to escalate towards homicide. Additionally, both Kivisto (2015) and Péloquin et al.’s (2024) findings include offenders resembling Holtzworth-Munroe et al.’s (2000) subtypes, as well as classifications unique to homicide offenders. Further investigation into homicidal offenders, possibly utilising psychosocial variables like those used to examine dysphoric/borderline offenders, could yield richer information into unique typologies and provide important insights into factors contributing to offending in this high-risk group.

### Cluster 2: GVA Offenders

The GVA cluster displayed highly general offending, seen through high medians in the largest range of crimes, against the highest numbers of victims. Aligning with these findings, previous literature identified GVA offenders as highly generalist, demonstrating antisocial characteristics and committing a wide range of frequent intra and extrafamilial offences (Petersson et al., 2019; Weber & Bouman, 2020; Wray et al., 2015). Although present study data cannot investigate personality, this offending pattern could indicate psychopathic and antisocial personality characteristics (Cunha et al., 2022; Hamberger & Langhinrichsen-Rohling, 2020), commonly seen in GVA offenders (Boyle et al., 2008; Holtzworth-Munroe & Stuart, 1994).

Contrary to previous literature, GVA offenders were not the most harmful cluster, as homicidal offenders caused significantly more harm. Although homicidal offenders were identified as a distinct subtype in this sample, further analyses may reveal they largely consist of offenders matching the GVA profile, as seen in Dixon et al.’s (2008) femicide offender study. Indeed, the present GVA cluster contained two offenders who had each committed one homicide, and were the only homicide offenders not placed in the homicidal subtype. This shows the distinction between GVA and homicidal offenders may be blurry. Nevertheless, GVA offenders’ higher harm scores compared to LLA and family-only align with previous literature (Peters et al., 2023; Serie et al., 2015; Thijssen & de Ruiter, 2011) determining GVA to be high-risk offenders, providing implications for prioritising offender response and rehabilitation (Cavanaugh & Gelles, 2005).

### Cluster 3: LLA Offenders

The LLA cluster showed a similar yet less severe offending pattern to GVA, matching previous literature, which consistently found LLA clustering between family-only and GVA (Graña et al., 2014; Weber & Bouman, 2020). In contrast, the LLA cluster displayed highest median scores for domestic violence and related offences. This may be due to the nature of the QPRIME database, which did not clearly identify which offences were related to DFV apart from index offences and offences connected to domestic violence applications or breaches of domestic violence orders. Categories such as homicide, assault, sexual, and weapons offences may also relate to DFV. In this case, LLA displaying higher rates of domestic violence and related offences may in fact indicate higher rates of less severe forms of DFV, like breaches of domestic violence orders, whereas GVA offenders perpetrated higher rates of more severe DFV (categorised in other offences like assaults), aligning with the literature (Graña et al., 2014; Weber & Bouman, 2020). Similarly, this subtype was responsible for significantly more harm than family-only offenders, and significantly less harm than GVA, matching previous literature identifying LLA as moderate risk (Llor-Esteban et al., 2016). Although not hypothesised, the identification of LLA offenders was not surprising.

### Cluster 4: Family-Only Offenders

Family-only offenders were characterised by the lowest levels of offending, with most relating to DFV. They aligned closely to the literature, with crimes restricted to intrafamilial DFV offences, of a lesser frequency and severity than other subtypes (Petersson & Strand, 2020; Petersson et al., 2019; Wray et al., 2015). This cluster comprised nearly half the sample, aligning with proportions in previous literature (Petersson & Strand, 2020). Domestic violence and related offences were their most common offence, and others present were typically common offences among all clusters that caused less harm, such as traffic/vehicle regulatory offences. Offences against justice procedures and government were slightly elevated compared to other offence types, but still lower than in other clusters. This may be because these offences can co-occur with DFV offences, such as resisting arrest, but more detailed data are needed to understand if this is the case. Harm scores were significantly lower than all other clusters, reflecting the lower risk of family-only offenders consistently seen in the literature (Llor-Esteban et al., 2016; Peters et al., 2023; Petersson & Strand, 2017; Serie et al., 2015; Thijssen & de Ruiter, 2011).

### Implications

#### Research

The study demonstrates Holtzworth-Munroe et al.’s (2000) family-only, LLA, and GVA offenders may be readily identified using only generality of offending through police data, which could allow future research to examine subtypes with more consistent methodologies and at a larger scale. Identifying clusters through offence data requires less one-on-one contact with offenders and less reliance on subjective measures known to introduce bias (Wray et al., 2015), making large-scale research more feasible and reliable, and addressing criticisms of inconsistent methodologies and underpowered samples (Alexander & Johnson, 2023).

The homicidal offenders subtype suggests these offenders do not neatly fit within other subtypes. As previous research identified domestic homicide offenders as heterogeneous in nature (Dixon et al., 2008; Kivisto, 2015; Péloquin et al., 2024; Vignola-Lévesque & Léveillée, 2021), examination into their own typologies and risk factors for homicide could assist future prevention efforts. As the present study did not allow for examination of psychopathologies, research using clinical measures to assess this could yield a richer understanding of this group.

All clusters contained a large age range with some distinctions between them; family-only being older on average, while GVA were younger. Additionally, homicidal offenders were the only grouping with no juvenile offenders. Although this paper did not assess ages in-depth, the literature generally identifies differing characteristics between DFV offenders in differing age ranges, particularly in comparing juvenile and adult offenders (e.g., Freeman, 2018). Further research examining age in more depth could provide a more refined understanding of offender types.

#### Practice

Offender subtyping at policing levels could support identification of high-risk offenders and triaging into appropriate interventions. The results demonstrate the utility of examining general offending in DFV perpetrators, suggesting that ignoring general offending histories could hinder identification of high-risk perpetrators. Additionally, outliers appearing at higher ends of offending and harm levels suggest a problematic subset of high-risk offenders could be identified by police systems flagging offenders with outlying offence histories (e.g., individuals in the top 5% of offending). However, as offending history is a static risk factor – meaning offenders who were once high-risk but have since desisted would still show extensive histories – an over-reliance on this measure could lead to incorrectly labelling perpetrators as high-risk. Identification of offender risk could assist with determining appropriate policing responses to DFV and concentrating resources on higher-risk offenders (Boxall et al., 2015b).

For intervention services, offender classification could assist understanding specific factors to target in rehabilitation, like substance use or social skills (Cantos et al., 2019), understanding which offenders will benefit most from programmes (Huss & Ralston, 2008), anticipating noncompliance and drop-out (Cantos et al., 2015, 2019), and enhancing risk assessment tools (Boxall et al., 2015b). Cantos et al. (2019) found GVA offenders were significantly more likely to reoffend and drop-out of treatments, but reoffending rates improved more when GVA completed treatment compared to family-only offenders. It was hypothesised that any level of treatment was effective for lowering recidivism in family-only, while completing treatment was more important for GVA. This demonstrates the need for increased concentration of resources allocated to higher-risk offenders.

### Limitations

Several limitations were identified based on the nature of the archival data. Firstly, data did not allow for identification of offender-victim relationships, meaning cases of IPV or other specific forms of DFV could not be reliably identified and examined separately. As Holtzworth-Munroe et al.’s (2000) IPV typologies were one of the most influential and well-backed theories, this was used as the basis for our research despite our sample comprising a broader group of DFV offenders. As 72% of Queensland DFV applications relate to IPV, this theory is still highly relevant for our sample (Queensland Courts, 2025). Although our sample likely comprises mostly IPV perpetrators, the fact that this cannot be accurately identified limits the findings’ interpretability.

Descriptions for offences were limited, and apart from indexes, domestic violence applications, and breaches of domestic violence orders, DFV offending could not be differentiated from general offences of that nature (e.g., discrimination between assaults on partners and on strangers). This limits inferences that can be made from clusters found in the dataset, however, meaningfully different clusters were still identifiable.

Due to the nature of policing data, any DFV cases not reported to the police were missing from the sample. This likely biases the data towards higher-risk offenders, and the proportion of family-only offenders are likely larger in community samples (Holtzworth-Munroe et al., 2000). However, this was seen mostly as a strength. Identifying high-risk offenders may be more important for response efforts, as they require more intervention (Cantos et al., 2019; Huss & Ralston, 2008).

Lastly, as data only included variables relating to offending generality, other characteristics commonly used to examine typologies such as psychopathology could not be examined. Although our methods can increase methodological consistency among research and limit social desirability biases (Alexander & Johnson, 2023; Wray et al., 2015), identification of clusters characterised largely through psychopathologies (e.g., dysphoric/borderline) cannot be made.

## Conclusion

Our study extended DFV typological literature by examining clusters of offenders at a large scale utilising policing data of offending histories. The four-cluster solution revealed subtypes of significantly differing harm risk that replicated Holtzworth-Munroe et al.’s (2000) GVA, LLA, and family-only typologies, with the addition of a novel fourth cluster: homicidal offenders. This demonstrated the uniqueness of these offenders and suggests further examination specific to homicide offenders is needed. The study demonstrated valid offender typologies could be identified at a large scale and at a policing level, providing important implications for future research methodologies as well as potential avenues to enhance policing risk assessment and concentration of resources, to address and reduce harm.

## Figures and Tables

**Appendix Figure A1.**
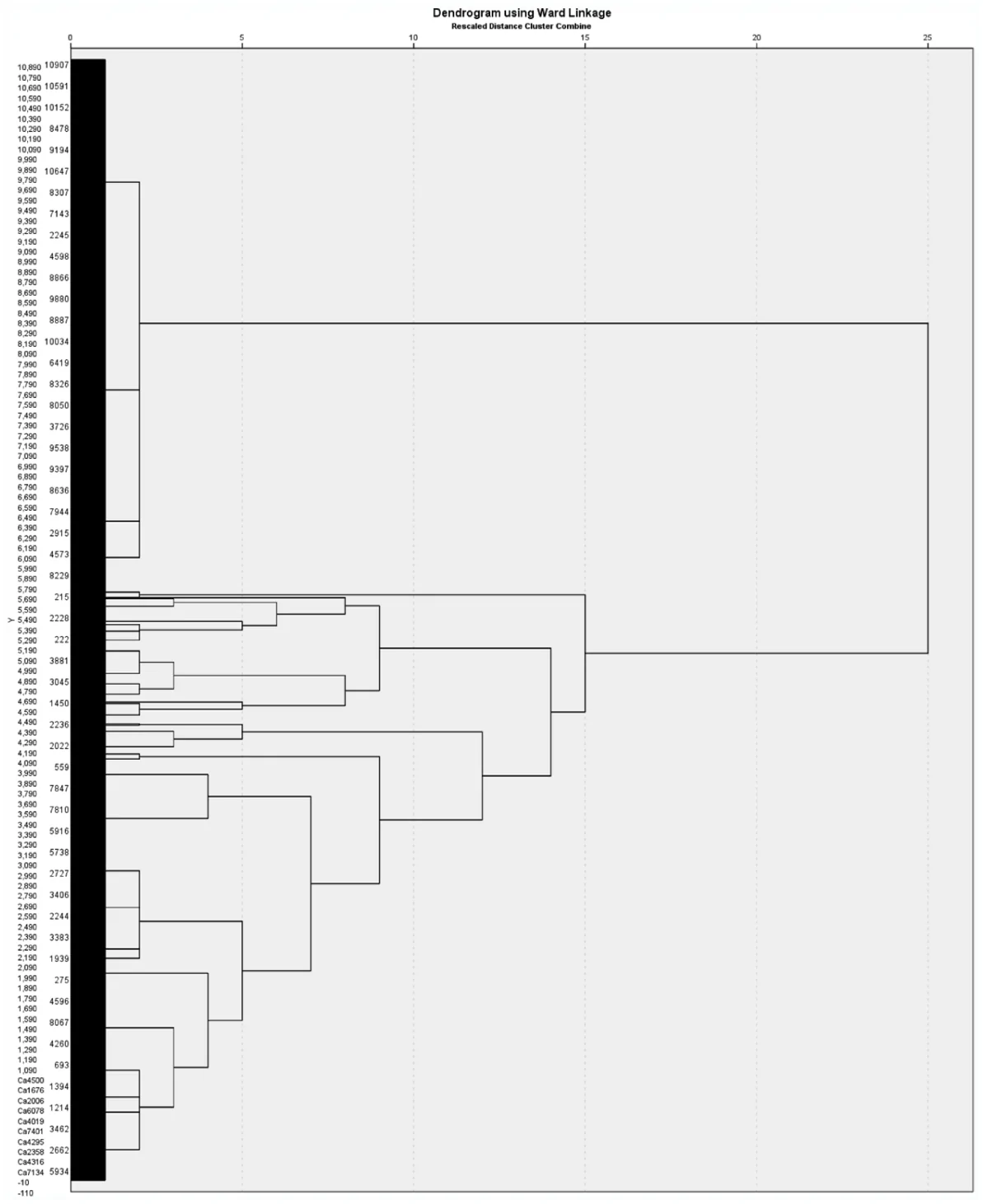
Hierarchical cluster analysis with ward’s linkage: dendrogram of relationships between clusters.

**Appendix Figure A2.**
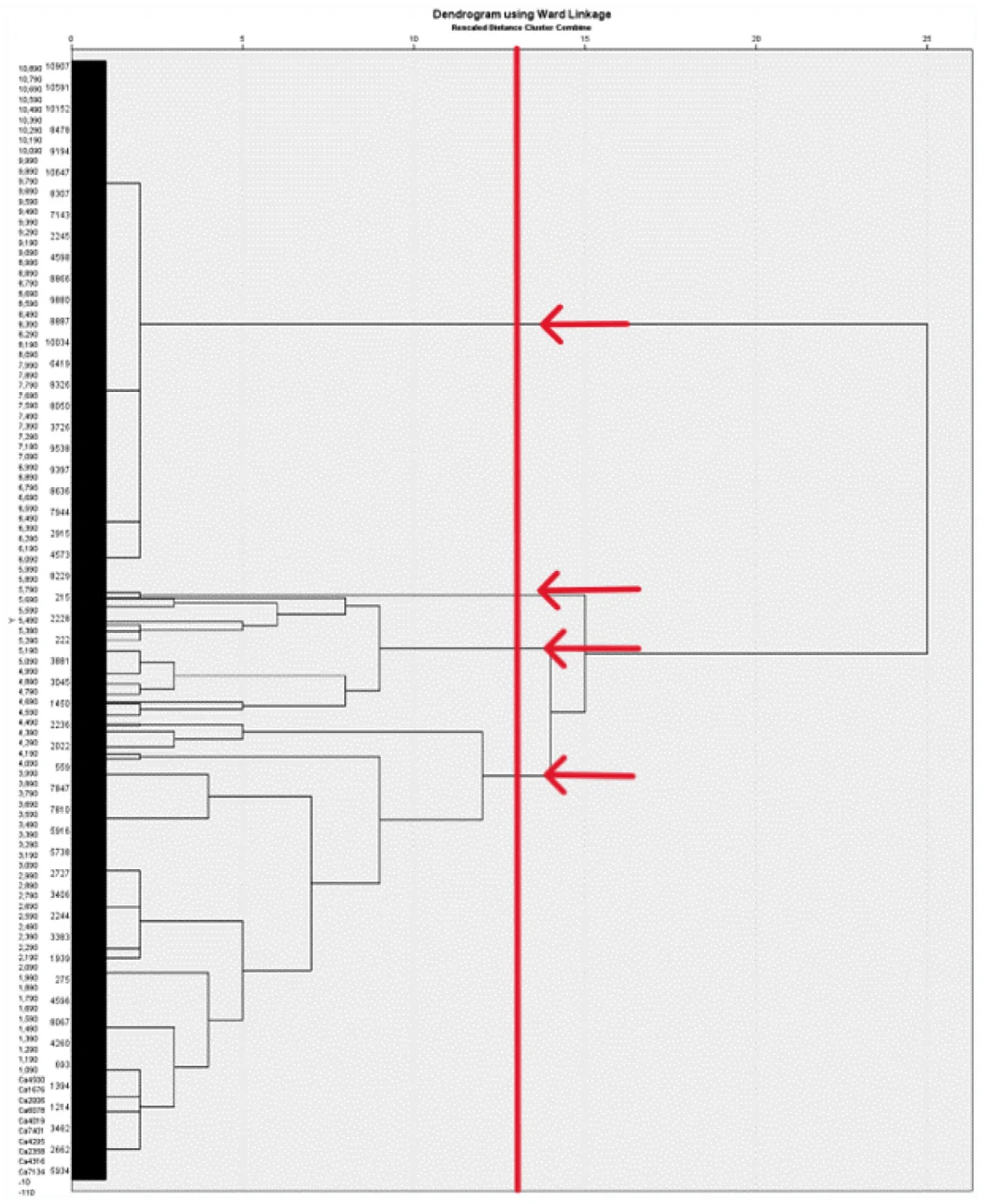
Hierarchical cluster analysis with ward’s linkage: dendrogram with ‘sliced’ final cluster solution. Red arrows indicate the number and placement of clusters revealed after ‘slicing’ the dendrogram.

**Appendix Figure B1.**
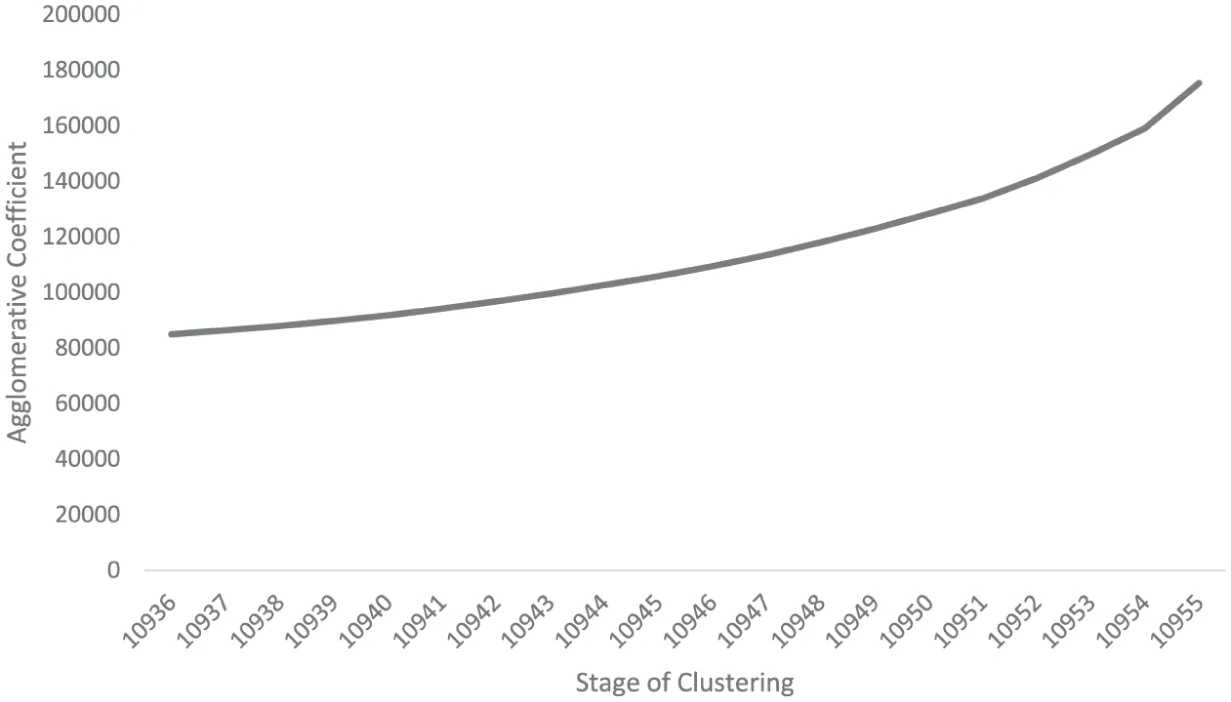
Line graph visualising cluster analysis agglomerative coefficients.

**Appendix Figure B2.**
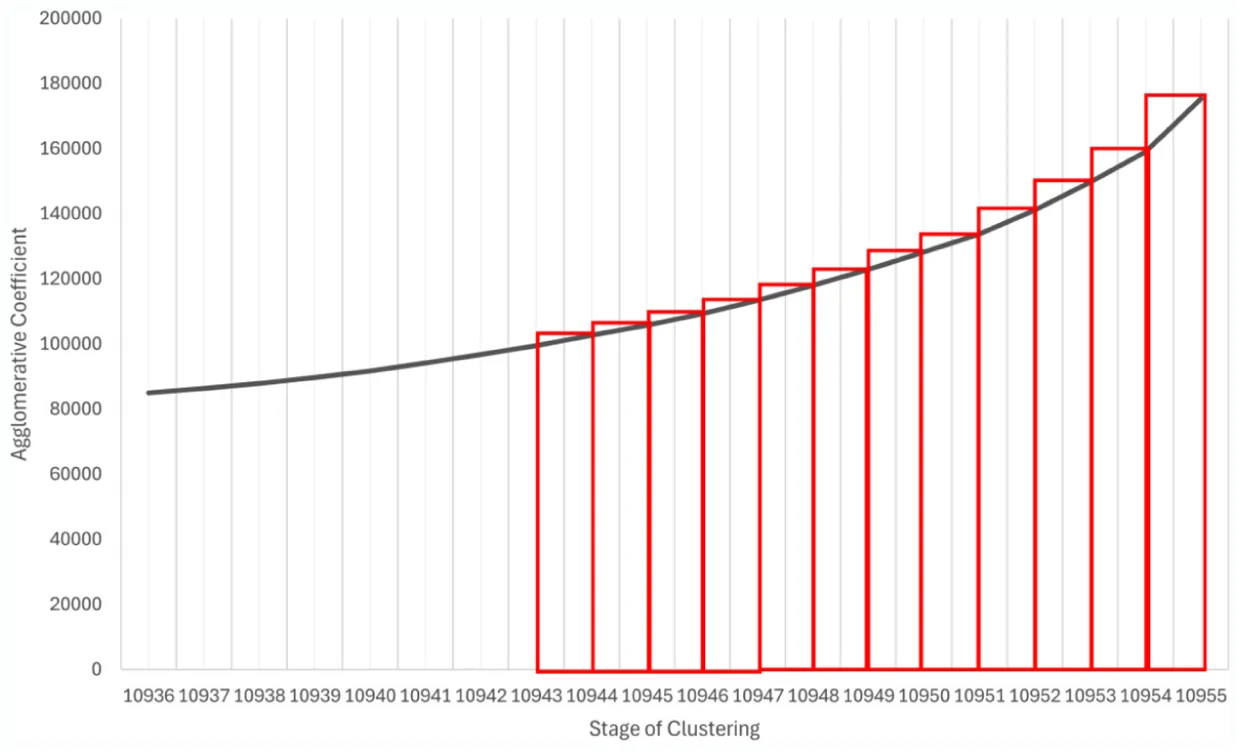
Agglomerative coefficients line graph with overlaying bar chart to aid visualisation.
